# T cell exhaustion in sepsis: mechanisms, biomarkers, and immune-reversal strategies

**DOI:** 10.3389/fimmu.2026.1897839

**Published:** 2026-09-03

**Authors:** Dongyang Li, Yuwei Wang, Jingren Li, Huiyuan Chen, Yi Cui, Wenjun Xia, Hao Wang

**Affiliations:** 1 Department of Critical Care Medicine, Qilu Hospital, Shandong University; Innovation Research Center for Sepsis and Multiple Organ Injury, Shandong University, Jinan, Shandong, China; 2 Department of Critical Care Medicine, Shandong Provincial Hospital Affiliated to Shandong First Medical University, Jinan, Shandong, China; 3 Department of Cardiology, Beijing Chest Hospital, Capital Medical University, Beijing, China; 4 Department of Cardiology, Beijing Tuberculosis and Thoracic Tumor Institute, Beijing, China; 5 Department of Critical Care Medicine, Qilu Hospital of Shandong University, Jinan, Shandong, China

**Keywords:** immune reversal, PD-1/PD-L1, sepsis, T-cell exhaustion, TIGIT

## Abstract

Sepsis is increasingly recognized as a dynamic immune disorder in which early hyperinflammation may coexist with or progress to profound immunosuppression. T-cell exhaustion is a central feature of this immune paralysis and is characterized by lymphopenia, impaired proliferation, reduced effector cytokine production, inhibitory receptor upregulation, metabolic dysfunction, and defective antibacterial immunity. Recent studies have expanded the mechanistic landscape of sepsis-induced T-cell exhaustion beyond the classical PD-1/PD-L1 axis. TIGIT-mediated suppression of CD4^+^ T-cell immunity, extracellular vesicle-associated PD-L1, TOX-independent exhaustion-like programming, dysregulated IL-17 production, mitochondrial dysfunction, and reduced glutaminase expression in CD4^+^ T cells have emerged as important mechanisms. These findings indicate that septic T-cell exhaustion is not a direct replica of exhaustion in cancer or chronic viral infection, but a context-dependent and potentially reversible immune dysfunction state. Biomarkers such as PD-1^+^CD3^+^ T cells, soluble PD-1/PD-L1, IL-7, TIGIT, EV-PD-L1, and GLS may support patient stratification and dynamic immune monitoring. Immune-reversal strategies, including PD-1/PD-L1 blockade, TIGIT inhibition, IL-7-based restoration, and metabolic reprogramming, require precise timing and individualized selection to restore host defense without aggravating inflammatory organ injury.

## Highlights

- Sepsis-induced T-cell exhaustion involves checkpoint activation, cytokine dysfunction, metabolic remodeling, and impaired antibacterial immunity.
- Recent studies highlight TIGIT, EV-PD-L1, TOX-independent programming, IL-17 dysregulation, and GLS reduction as emerging mechanisms.
- Immune-reversal strategies require biomarker-guided timing and patient stratification rather than non-specific immune stimulation.

## 1 Introduction

Sepsis is a life-threatening syndrome caused by a dysregulated host response to infection and remains a major cause of mortality in intensive care units worldwide (1–3). Traditionally, sepsis has been viewed primarily as a hyperinflammatory disorder characterized by excessive cytokine release, endothelial injury, coagulation abnormalities, and progressive organ dysfunction. This concept has greatly shaped early therapeutic strategies aimed at controlling overwhelming inflammation. However, accumulating evidence indicates that the immune response in sepsis is not simply defined by uncontrolled inflammation (4–8). Instead, sepsis involves a complex and dynamic immune trajectory in which early inflammatory activation often coexists with, or is followed by, profound immunosuppression. Many patients who survive the initial inflammatory phase develop persistent immune dysfunction, characterized by impaired pathogen clearance, increased susceptibility to secondary infections, viral reactivation, prolonged intensive care unit stay, and late mortality. Therefore, understanding sepsis as a biphasic or mixed immune disorder is essential for developing more precise immunomodulatory strategies.

Among the immune alterations observed during sepsis, T-cell dysfunction represents one of the most important features of sepsis-induced immunosuppression. Both CD4^+^ and CD8^+^ T cells may undergo marked numerical and functional changes after septic insult (9–12). Lymphopenia is frequently observed and can result from apoptosis, impaired thymic output, altered peripheral survival, and redistribution of lymphocytes into tissues (13, 14). Beyond cell loss, surviving T cells often display reduced proliferative capacity, impaired antigen responsiveness, altered cytokine production, and defective effector function. These changes compromise the ability of the host to mount effective adaptive immune responses against primary and secondary infections. In parallel, septic T cells may upregulate multiple inhibitory receptors, including programmed cell death protein 1 (PD-1), T-cell immunoreceptor with Ig and ITIM domains (TIGIT), T-cell immunoglobulin and mucin domain-containing protein 3 (TIM-3), lymphocyte activation gene 3 (LAG-3), and 2B4. Programmed death-ligand 1 (PD-L1), which is expressed by antigen-presenting cells, myeloid cells, endothelial cells, tissue cells, and extracellular vesicles, may further contribute to checkpoint-mediated T-cell suppression (15–17). The sustained or coordinated expression of these molecules resembles the exhausted T-cell phenotype described in chronic viral infection and cancer, supporting the concept that T-cell exhaustion is a central component of sepsis-associated immune paralysis.

Nevertheless, T-cell exhaustion in sepsis should not be interpreted as a direct copy of classical exhaustion programs observed in tumors or chronic infections (18, 19). In cancer and chronic viral infection, T-cell exhaustion usually develops under conditions of prolonged antigen stimulation and is associated with relatively stable transcriptional and epigenetic remodeling (20). By contrast, sepsis is an acute and highly dynamic syndrome shaped by fluctuating pathogen burden, systemic inflammation, tissue damage, metabolic stress, organ dysfunction, and therapeutic interventions (21). As a result, sepsis-induced T-cell exhaustion may represent an “exhaustion-like” dysfunctional state rather than a fixed lineage program. This distinction is important because it suggests that biomarkers and therapeutic strategies developed in oncology or chronic infection may not be directly transferable to sepsis without considering disease stage, immune phenotype, infection control, and organ injury status (22).

Recent studies have expanded the mechanistic landscape of sepsis-induced T-cell exhaustion beyond the classical PD-1/PD-L1 axis (15, 22). Emerging mechanisms include TIGIT-mediated CD4^+^ T-cell suppression, EV-associated PD-L1, TOX-independent exhaustion-like programming, IL-17-related metabolic dysfunction, mitochondrial stress, and impaired glutamine metabolism. These mechanisms are discussed in detail in the following sections (16). Together, they support the concept that sepsis-induced T-cell exhaustion is a dynamic, context-dependent, and potentially reversible immune dysfunction state rather than a direct replica of classical exhaustion in cancer or chronic viral infection.

In this review, we summarize recent advances in understanding T-cell exhaustion in sepsis, with a focus on checkpoint signaling, TOX-independent exhaustion-like programming, metabolic dysfunction, biomarker discovery, and immune-reversal strategies. We argue that sepsis-induced T-cell exhaustion should be viewed as a dynamic and context-dependent immune state rather than a uniform terminal phenotype. A better understanding of this state may support the development of stage-specific biomarkers and personalized immunotherapies aimed at restoring protective immunity without aggravating harmful inflammation.

## 2 Phenotypic features of sepsis-induced t-cell exhaustion

### 2.1 Lymphopenia and impaired T-cell effector function

Phenotypically, sepsis-induced T-cell exhaustion is characterized by both quantitative and qualitative defects in adaptive immunity. One of the most prominent changes is lymphopenia, which affects both CD4^+^ and CD8^+^ T-cell compartments (23–25). During the acute phase of sepsis, extensive lymphocyte apoptosis can rapidly reduce circulating T-cell numbers. This loss is particularly important because T cells are required for pathogen-specific immunity, coordination of innate immune responses, and long-term immune surveillance. In patients who survive the early inflammatory phase, incomplete restoration of the T-cell pool may contribute to prolonged immune paralysis, recurrent infection, and poor clinical recovery. Therefore, T-cell exhaustion in sepsis should not be viewed only as a molecular phenotype defined by inhibitory receptor expression, but also as a broader state of numerical depletion and impaired immune competence.

Beyond reduced cell numbers, surviving T cells frequently show impaired effector function (17). Septic T cells often exhibit decreased proliferative capacity after antigenic or mitogenic stimulation, indicating that these cells fail to expand efficiently when immune activation is required (26). This defect may compromise the host response to persistent pathogens and secondary infections (21). In parallel, cytokine production is frequently altered. CD4^+^ T cells may produce lower levels of interleukin-2 (IL-2), which is essential for T-cell expansion and survival, while both CD4^+^ and CD8^+^ T cells may show impaired interferon-γ (IFN-γ) production, weakening macrophage activation, intracellular pathogen control, and antibacterial immunity (17, 26, 27). Reduced production of these effector cytokines reflects a loss of functional fitness rather than simple changes in T-cell abundance.

Importantly, sepsis-induced T-cell dysfunction is not limited to conventional exhaustion markers. It also involves abnormalities in activation status, differentiation state, cytokine balance, metabolic activity, and memory-like remodeling. Some T cells acquire an activated or memory-like phenotype but remain functionally compromised, suggesting a dissociation between phenotypic activation and effective immune protection. This distinction is critical because an increase in activation-associated surface markers does not necessarily indicate preserved antimicrobial function. In the post-septic immune environment, T cells may simultaneously display features of activation, exhaustion, metabolic stress, and impaired effector capacity (28, 29). Thus, the term “T-cell exhaustion” in sepsis should be used cautiously to describe a complex and dynamic dysfunction state rather than a single terminal differentiation program.

The clinical consequence of this dysfunctional phenotype is particularly evident during secondary infections. Patients with sepsis often remain vulnerable to nosocomial infections, opportunistic pathogens, and viral reactivation even after initial hemodynamic stabilization. Experimental studies also show that post-septic hosts may fail to generate effective T-cell responses against secondary infectious challenges. This impaired recall or secondary response suggests that sepsis disrupts not only immediate effector immunity but also the capacity of the adaptive immune system to recover and respond to new antigenic threats. Therefore, lymphopenia, defective proliferation, reduced IL-2 and IFN-γ production, and impaired secondary immune responses together define the functional basis of sepsis-induced T-cell exhaustion.

### 2.2 Inhibitory receptor upregulation: PD-1, PD-L1, TIGIT, TIM-3, LAG-3 and 2B4

A second major phenotypic feature of sepsis-induced T-cell exhaustion is the increased expression of inhibitory immune checkpoint molecules (22, 30–32). Among these, the PD-1/PD-L1 axis is the most extensively studied (22, 33). PD-1 is expressed on activated and dysfunctional T cells, whereas PD-L1 can be expressed by antigen-presenting cells, myeloid cells, endothelial cells, tissue cells, and potentially extracellular vesicles. Engagement of PD-1 by PD-L1 suppresses T-cell receptor signaling, limits proliferation, reduces cytokine production, and promotes an immunosuppressive state (27, 34). In sepsis, increased PD-1 expression on T cells and increased PD-L1 expression in the immune environment have been associated with impaired T-cell function and adverse outcomes, making this axis a central marker and mediator of sepsis-associated immune paralysis.

Functionally, PD-1/PD-L1 signaling is not merely a phenotypic marker but also a regulatory pathway that can restrain T-cell activation during sepsis. Its timing-dependent therapeutic implications are discussed in detail in Section 5.1. This is particularly relevant for defining exhaustion-related phenotypes because PD-1 expression may reflect different biological states depending on disease stage, infection burden, and inflammatory context. In addition to PD-1/PD-L1, other inhibitory receptors are increasingly recognized as part of the exhausted T-cell phenotype in sepsis. T-cell immunoglobulin and mucin domain-containing protein 3 (TIM-3), lymphocyte activation gene 3 (LAG-3), and 2B4 may be co-expressed with PD-1 on dysfunctional T cells. Their combined expression suggests a more severe or persistent state of T-cell impairment. In chronic viral infection and cancer, co-expression of multiple inhibitory receptors is often associated with deeper exhaustion and reduced reversibility. A similar principle may apply to sepsis, although the kinetics and stability of these receptors may differ because sepsis is an acute and fluctuating immune disorder. Therefore, evaluating a single checkpoint molecule may be insufficient. A broader inhibitory receptor profile may better capture the heterogeneity and severity of T-cell dysfunction in septic patients.

TIGIT has also emerged as an inhibitory receptor associated with sepsis-induced T-cell dysfunction (29, 30). Together with PD-1, TIM-3, LAG-3, and 2B4, TIGIT may help define a broader checkpoint-positive phenotype in septic T cells. Its functional role in CD4^+^ T-cell antibacterial immunity is discussed in detail in Section 3.2. The expansion of the checkpoint landscape has several implications. First, sepsis-induced T-cell exhaustion should be defined by an integrated phenotypic profile rather than by PD-1 expression alone. Second, different inhibitory receptors may regulate distinct aspects of T-cell dysfunction. For example, PD-1/PD-L1 may primarily suppress T-cell receptor signaling and proliferation, whereas TIGIT may further restrain CD4^+^ T-cell antibacterial immunity. TIM-3, LAG-3, and 2B4 may mark additional layers of dysfunction or identify T-cell subsets with more profound exhaustion. Third, inhibitory receptor expression must be interpreted in relation to disease stage. Early after infection, checkpoint expression may serve as a physiological brake to limit tissue-damaging inflammation. However, persistent or excessive checkpoint signaling may contribute to immune paralysis, defective microbial clearance, and susceptibility to secondary infections.

**Figure 1:**
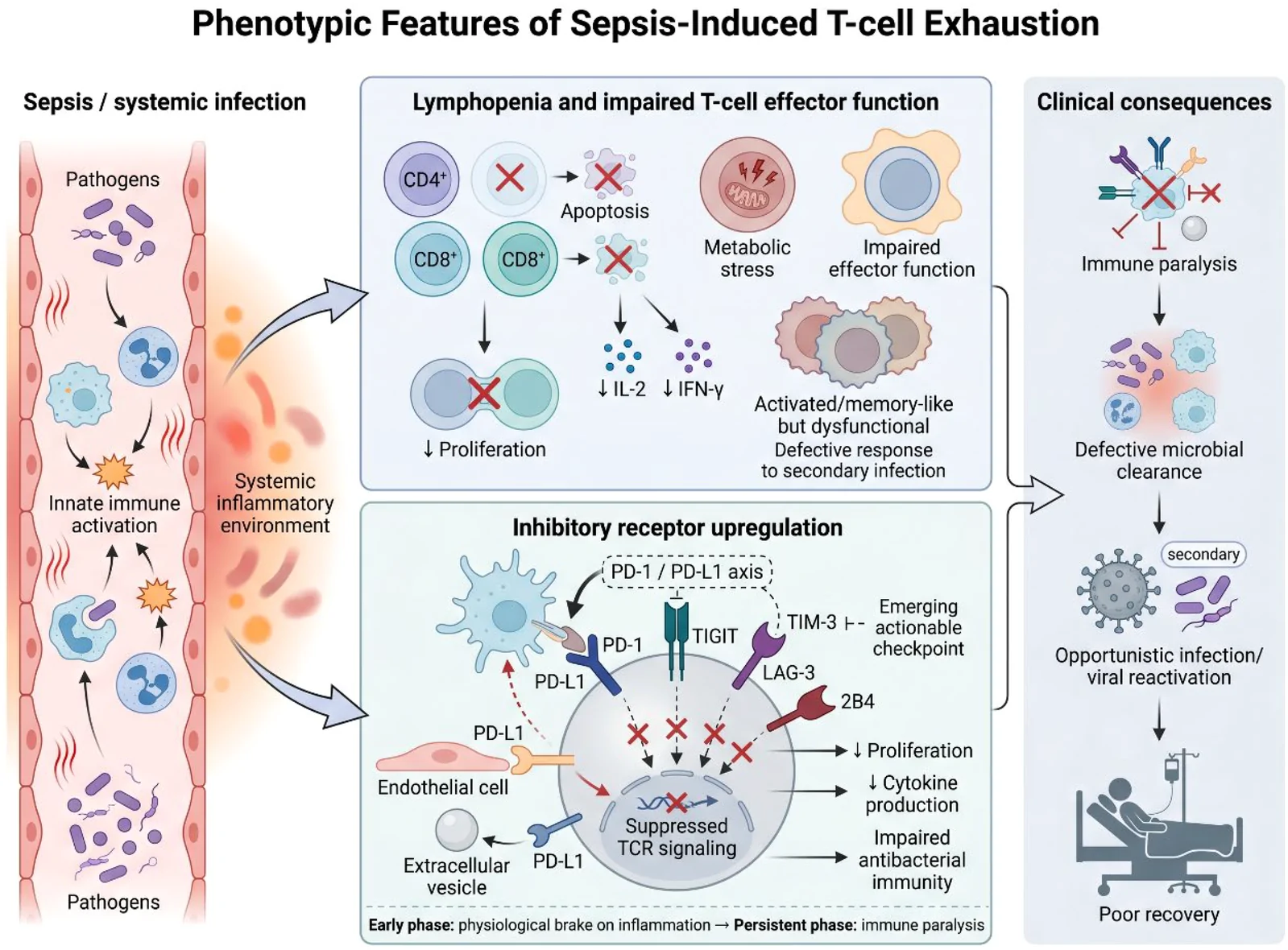
Phenotypic features of sepsis-induced T-cell exhaustion. Sepsis-induced T-cell exhaustion is characterized by lymphopenia, CD4^+^ and CD8^+^ T-cell apoptosis, impaired proliferation, reduced IL-2 and IFN-γ production, and defective secondary immune responses. In parallel, dysfunctional T cells upregulate inhibitory receptors, including PD-1, TIGIT, TIM-3, LAG-3, and 2B4, while PD-L1-expressing immune cells or extracellular vesicles further suppress antibacterial immunity.

Taken together, the phenotypic features of sepsis-induced T-cell exhaustion include lymphopenia, impaired proliferation, reduced effector cytokine production, defective responses to secondary infection, and increased expression of multiple inhibitory receptors. These features support the concept that T-cell exhaustion in sepsis is a multidimensional immune state involving cell loss, functional suppression, altered differentiation, and checkpoint-mediated inhibition. Recent studies on PD-1/PD-L1 and TIGIT further indicate that inhibitory receptors are not only biomarkers of dysfunction but also functional regulators of impaired antibacterial immunity (33–36). This phenotypic framework provides the basis for understanding the mechanisms, biomarkers, and immune-reversal strategies discussed in the following sections. **Figure 1** summarizes the major phenotypic features of sepsis-induced T-cell exhaustion, emphasizing that this state involves both numerical T-cell depletion and qualitative immune dysfunction. It also highlights that checkpoint upregulation, especially PD-1/PD-L1 and TIGIT, cooperates with impaired cytokine production and defective proliferation to promote immune paralysis and secondary infection susceptibility.

## 3 Mechanistic programs driving T-cell exhaustion in sepsis

### 3.1 PD-1/PD-L1 signaling as a central checkpoint axis

PD-1/PD-L1 signaling represents one of the most established immune checkpoint pathways involved in sepsis-induced T-cell exhaustion (37–39). PD-1 is commonly upregulated on activated or dysfunctional T cells after septic insult, whereas PD-L1 can be expressed by multiple cell types within the inflammatory and immunosuppressive milieu, including monocytes, macrophages, dendritic cells, neutrophils, endothelial cells, epithelial cells, and tissue-resident immune cells. The broad distribution of PD-L1 suggests that T-cell inhibition in sepsis is not restricted to classical antigen-presenting cell–T-cell interactions but may occur across multiple immune and tissue compartments. This is particularly relevant in sepsis, where systemic inflammation, endothelial activation, microbial products, and tissue injury collectively reshape immune checkpoint expression.

In sepsis, PD-1/PD-L1 signaling should be interpreted as a dynamic regulatory axis rather than a uniformly pathogenic checkpoint (22, 40). Depending on disease stage, inflammatory intensity, pathogen burden, and organ injury, this pathway may either restrain excessive inflammation or contribute to pathological T-cell dysfunction. Therefore, the significance of PD-1/PD-L1 expression should be evaluated in relation to immune phase and clinical context, while the therapeutic implications of PD-1/PD-L1 blockade are discussed in Section 5.1. EV-associated PD-L1 represents a circulating extension of the PD-1/PD-L1 checkpoint axis and may contribute to systemic T-cell suppression during sepsis.

Together, these findings position PD-1/PD-L1 signaling as a central checkpoint axis in sepsis-induced T-cell exhaustion. However, they also highlight that this pathway is dynamic rather than uniformly harmful. The challenge is not simply to block PD-1/PD-L1, but to identify when this axis shifts from a protective anti-inflammatory brake to a driver of pathological immune paralysis. This distinction has important implications for biomarker-guided therapy and for the design of immune-reversal strategies in sepsis.

### 3.2 TIGIT-mediated suppression of CD4^+^ T-cell antibacterial immunity

Although PD-1/PD-L1 remains the best-characterized checkpoint pathway in sepsis, other inhibitory receptors are increasingly recognized as important contributors to T-cell dysfunction. TIGIT has recently emerged as a particularly relevant molecule. TIGIT is an inhibitory immune checkpoint receptor expressed on activated T cells, regulatory T cells, natural killer cells, and other lymphocyte subsets. Through interactions with ligands such as CD155 and CD112, TIGIT can suppress T-cell activation, reduce cytokine production, and promote immune tolerance. In cancer immunology, TIGIT has been widely studied as a mediator of T-cell exhaustion and immune escape. Its role in sepsis, however, has only recently begun to receive attention.

Zhong et al. provides important mechanistic evidence that TIGIT is not only a phenotypic marker but also a functional regulator of antibacterial immunity during sepsis (16). In polymicrobial sepsis models, TIGIT expression was increased on immune cells, and TIGIT signaling limited CD4^+^ T-cell proliferation and antibacterial cytokine responses. Conversely, TIGIT deficiency or antibody-mediated inhibition improved CD4^+^ T-cell function and enhanced host defense. These findings suggest that TIGIT participates directly in the suppression of protective adaptive immunity during sepsis. The relevance of TIGIT is particularly important for CD4^+^ T-cell biology. CD4^+^ T cells coordinate multiple arms of antibacterial immunity, including macrophage activation, B-cell help, cytokine production, and the orchestration of innate–adaptive immune crosstalk. When TIGIT signaling suppresses CD4^+^ T-cell activation or proliferation, the consequences may extend beyond the T-cell compartment itself. Reduced CD4^+^ T-cell help may impair pathogen clearance, weaken myeloid cell activation, and contribute to persistent infection. Therefore, TIGIT-mediated suppression may represent a nodal mechanism linking checkpoint signaling to systemic immune paralysis.

TIGIT may also interact with the PD-1/PD-L1 axis to form a broader inhibitory receptor network (41, 42). In many immune contexts, exhausted T cells do not express a single inhibitory receptor but instead co-express multiple checkpoints (18, 43). This co-expression can impose layered inhibition on TCR signaling, cytokine production, metabolic fitness, and tissue persistence. In sepsis, PD-1 and TIGIT may regulate overlapping but non-identical aspects of T-cell dysfunction (15, 44). PD-1 may predominantly suppress TCR-driven activation and proliferation, whereas TIGIT may further restrain CD4^+^ T-cell antibacterial effector programs. This raises the possibility that combined or sequential targeting of checkpoint pathways may be more effective than single-agent blockade in selected immunosuppressed patients. However, such strategies would require careful timing and immune monitoring to avoid aggravating inflammation or tissue injury.

Thus, TIGIT represents a new and promising mechanism in the field of sepsis-induced T-cell exhaustion. Its inclusion broadens the checkpoint landscape beyond PD-1/PD-L1 and supports a more nuanced view of immune suppression in sepsis, in which multiple inhibitory receptors cooperate to constrain adaptive immunity.

### 3.3 TOX-independent exhaustion-like programming in sepsis

A key unresolved question is whether T-cell exhaustion in sepsis follows the same transcriptional logic as exhaustion in chronic viral infection or cancer. In those settings, persistent antigen exposure drives a relatively stable exhausted state characterized by progressive loss of effector function, sustained inhibitory receptor expression, metabolic adaptation, and epigenetic remodeling. The transcription factor thymocyte selection-associated high mobility group box protein TOX has been identified as a central regulator of exhausted CD8^+^ T-cell differentiation in chronic viral infection and cancer, where it contributes to transcriptional and epigenetic exhaustion programs (45–47). Because septic T cells also upregulate inhibitory receptors and lose effector function, it might be tempting to assume that sepsis-induced exhaustion is controlled by the same TOX-dependent program. However, Qin et al. showed that chronic sepsis can induce T cells with exhaustion-associated features, including increased expression of PD-1, LAG-3, and TIM-3; nevertheless, TOX deficiency did not prevent or fully reverse the development of sepsis-induced T-cell exhaustion (47). This finding indicates that, despite phenotypic overlap with classical exhausted T cells, septic T cells may not depend on the canonical TOX-centered transcriptional program. This observation is conceptually important because it separates sepsis-induced T-cell exhaustion from canonical exhaustion programs described in chronic viral infection and cancer. Therefore, sepsis-associated T-cell exhaustion should be viewed as an exhaustion-like immune dysfunction state rather than a direct copy of tumor-associated exhausted T cells. The septic immune environment differs fundamentally from the tumor microenvironment or chronic viral infection. Sepsis is characterized by abrupt systemic inflammation, massive cell death, lymphocyte apoptosis, metabolic stress, tissue hypoxia, microbial translocation, antibiotic exposure, organ dysfunction, and fluctuating antigenic stimulation (48–51). These factors may generate a hybrid T-cell state that combines features of activation, apoptosis resistance or susceptibility, metabolic exhaustion, cytokine imbalance, and checkpoint-mediated inhibition.

The TOX-independent nature of sepsis-associated T-cell dysfunction also has therapeutic implications. If septic T-cell exhaustion does not rely on the same transcriptional program as tumor-induced exhaustion, then therapies designed to reinvigorate tumor-infiltrating T cells may not produce the same effects in sepsis. For example, checkpoint blockade may only partially restore function if the underlying dysfunction is driven by metabolic collapse, cytokine deprivation, apoptosis, or systemic inflammatory damage. Similarly, strategies targeting transcriptional regulators of exhaustion may be less effective unless they are adapted to the acute and dynamic biology of sepsis. Future studies should therefore define the specific transcriptional, epigenetic, and metabolic circuits that distinguish sepsis-induced T-cell dysfunction from other exhausted states.

### 3.4 Metabolic reprogramming of CD4^+^ T cells: core pathways and broader metabolic context

T-cell exhaustion in sepsis is not only controlled by inhibitory receptors and transcriptional programs; it is also deeply shaped by metabolic reprogramming. T-cell activation requires coordinated metabolic transitions. Naïve T cells rely mainly on oxidative phosphorylation and fatty acid oxidation, whereas activated effector T cells increase glycolysis, amino acid uptake, mitochondrial activity, and biosynthetic metabolism to support clonal expansion and cytokine production. In sepsis, this metabolic coordination is disrupted. T cells may receive inflammatory and antigenic signals but fail to generate the metabolic fitness needed for sustained protective immunity (52–54). In this review, we focus primarily on mitochondrial dysfunction, glycolytic remodeling, and glutamine metabolism for two reasons. First, these pathways are directly linked to the bioenergetic requirements of CD4^+^ T-cell activation, proliferation, cytokine production, and survival. Mitochondria provide oxidative phosphorylation, redox control, and apoptotic regulation; glycolysis supports rapid effector activation; and glutamine metabolism supplies anaplerotic and biosynthetic substrates required for sustained T-cell responses. Second, recent sepsis-related studies have provided more direct evidence connecting these pathways with post-septic CD4^+^ T-cell dysfunction, impaired secondary immune responses, dysregulated IL-17 production, and reduced glutaminase expression. Therefore, these pathways were selected as the central focus of this section, not because other metabolic programs are unimportant, but because they currently have the most direct relevance to exhaustion-like CD4^+^ T-cell dysfunction in sepsis.

Assis et al. provides important evidence that CD4^+^ T cells undergo persistent metabolic and functional remodeling after sepsis (55). In this context, “dysregulated IL-17 production” does not simply refer to increased or decreased IL-17 levels. Rather, it denotes a maladaptive IL-17 response in post-septic CD4^+^ T cells, in which IL-17 production becomes uncoupled from effective antimicrobial protection and is associated with impaired responses to secondary infection. In the post-septic state, CD4^+^ T cells exhibited mitochondrial dysfunction and enhanced glycolytic activity, yet these metabolic changes did not translate into effective immune protection. Instead, they were associated with dysregulated interleukin-17 production and impaired responses to secondary pulmonary infection. This finding suggests that increased glycolysis alone should not be interpreted as effective T-cell activation. In sepsis, metabolic rewiring may become maladaptive, generating inflammatory imbalance while failing to restore pathogen-specific immunity.

Mitochondrial dysfunction is particularly relevant to T-cell exhaustion. Mitochondria regulate ATP production, reactive oxygen species signaling, calcium homeostasis, apoptosis, and metabolic flexibility (56, 57). When mitochondrial function is impaired, T cells may lose the ability to sustain proliferation, produce cytokines, and survive during immune challenge. In post-septic T cells, mitochondrial defects may therefore contribute to both reduced effector capacity and increased vulnerability to subsequent infection (55). At the same time, enhanced glycolysis may reflect a compensatory response to mitochondrial injury rather than a marker of productive immune activation. This mismatch between metabolic activation and functional output may be a defining feature of sepsis-induced T-cell dysfunction. Glutamine metabolism represents another metabolic layer of sepsis-induced CD4^+^ T-cell dysfunction. In post-septic immune stress, reduced GLS expression may indicate impaired glutamine-dependent metabolic fitness, limiting mitochondrial anaplerosis, redox control, and biosynthetic support required for T-cell proliferation and cytokine production (58).

Taken together, metabolic reprogramming provides a mechanistic bridge between systemic inflammation and adaptive immune failure. Sepsis exposes T cells to hypoxia, nutrient fluctuations, inflammatory cytokines, oxidative stress, and tissue-derived danger signals. These pressures reshape mitochondrial function, glycolysis, amino acid metabolism, lipid utilization, redox balance, and cytokine output. As a result, T cells may enter a state in which they are phenotypically activated, metabolically stressed, and functionally ineffective. Although mitochondrial dysfunction, glycolytic remodeling, and glutamine metabolism are emphasized here because of their direct relevance to CD4^+^ T-cell dysfunction in sepsis, a broader metabolic network involving other amino acids and metabolic intermediates should be considered in future studies. This metabolic perspective broadens the definition of sepsis-induced T-cell exhaustion and supports the development of immune-reversal strategies that combine checkpoint modulation with metabolic restoration.

**Figure 2:**
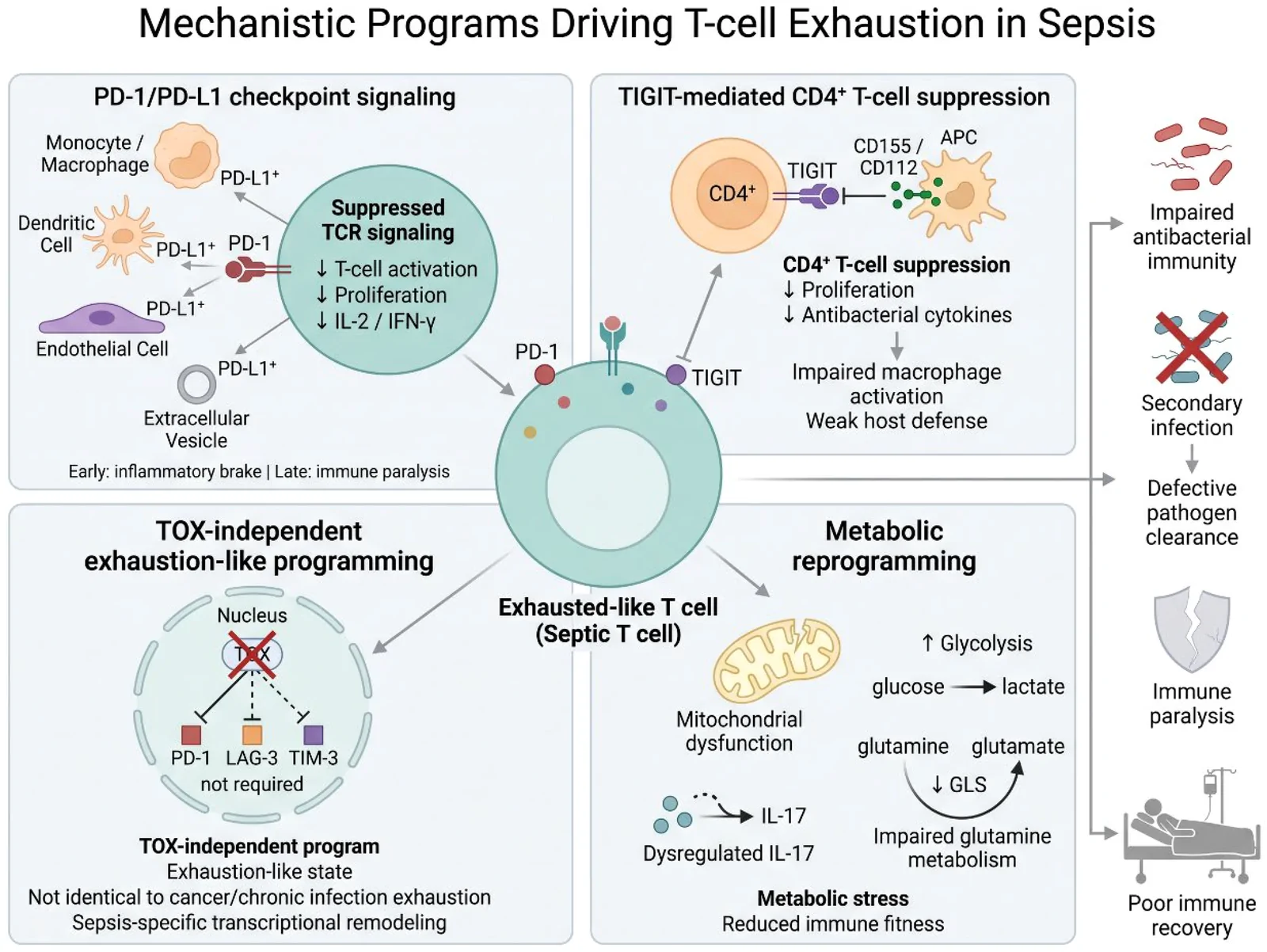
Mechanistic programs driving T-cell exhaustion in sepsis. Sepsis-induced T-cell exhaustion is driven by interconnected mechanisms, including PD-1/PD-L1 checkpoint signaling, EV-associated PD-L1-mediated suppression, TIGIT-dependent inhibition of CD4^+^ T-cell antibacterial immunity, TOX-independent exhaustion-like programming, and metabolic reprogramming characterized by mitochondrial dysfunction, increased glycolysis, reduced GLS, impaired glutamine metabolism, and dysregulated IL-17 production.

Overall, the mechanisms driving T-cell exhaustion in sepsis are multidimensional. PD-1/PD-L1 signaling imposes a central inhibitory checkpoint, including systemic suppression mediated by EV-associated PD-L1. TIGIT adds an emerging layer of CD4^+^ T-cell-specific antibacterial suppression. TOX-independent programming distinguishes sepsis-associated exhaustion-like states from classical exhaustion in cancer and chronic infection. Finally, metabolic reprogramming involving mitochondrial dysfunction, glycolytic imbalance, IL-17 dysregulation, and impaired glutamine metabolism provides a deeper explanation for why septic T cells often fail to recover full immune competence. These mechanisms collectively define sepsis-induced T-cell exhaustion as a dynamic, context-dependent, and potentially reversible immune dysfunction state. **Figure 2** illustrates that T-cell exhaustion in sepsis is not controlled by a single checkpoint pathway, but by coordinated checkpoint, transcriptional, and metabolic programs. It highlights that septic T cells may acquire an exhaustion-like state distinct from classical TOX-dependent exhaustion in cancer or chronic viral infection.

## 4 Biomarkers for identifying exhausted or dysfunctional T cells in sepsis

The clinical translation of sepsis-induced T-cell exhaustion requires reliable biomarkers that can identify patients with adaptive immune dysfunction, predict prognosis, and guide immune-reversal therapies. Conventional biomarkers used in sepsis, such as C-reactive protein, procalcitonin, lactate, white blood cell count, and inflammatory cytokines, mainly reflect infection burden, systemic inflammation, tissue hypoperfusion, or organ injury (59–62). Although these indicators are useful for diagnosis and risk assessment, they do not directly capture the functional state of T cells. Because sepsis is characterized by dynamic transitions between hyperinflammation and immunosuppression, biomarkers that reflect T-cell exhaustion or dysfunction may provide additional information beyond traditional inflammatory or organ failure markers. Ideally, such biomarkers should be measurable using peripheral blood samples, reproducible across time points, biologically linked to immune paralysis, and useful for selecting patients who may benefit from immunostimulatory treatment. In this context, peripheral blood biomarkers include not only soluble plasma or serum molecules, such as soluble PD-1, soluble PD-L1, and IL-7, but also immune-cell phenotypes measured in circulating leukocytes, such as PD-1^+^CD3^+^ T cells, TIGIT^+^ T cells, and metabolically altered CD4^+^ T-cell subsets. Therefore, cellular markers detected by flow cytometry or related single-cell assays should be considered part of peripheral blood immune monitoring rather than separate from peripheral blood biomarkers.

### 4.1 PD-1^+^CD3^+^ T cells as prognostic biomarkers

Among peripheral blood cellular biomarkers, PD-1^+^CD3^+^ T cells represent one of the most clinically accessible indicators of sepsis-associated T-cell dysfunction (63). CD3 identifies the total T-cell compartment, whereas PD-1 expression reflects inhibitory checkpoint activation and potential functional impairment (15, 27). Flow cytometric measurement of PD-1^+^CD3^+^ T cells can therefore provide a direct cellular readout of exhausted or suppressed adaptive immunity. Compared with soluble inflammatory mediators, this marker is more closely related to the immune status of T cells themselves and may help distinguish patients with predominant immune paralysis from those with mainly inflammatory activation (22, 63).

Wang et al. provides important clinical evidence supporting this concept (63). In this patient cohort, a higher proportion of PD-1^+^CD3^+^ T cells was associated with increased 30-day mortality. Importantly, PD-1^+^CD3^+^ T cells showed prognostic value beyond conventional clinical severity scores such as SOFA and APACHE II. The integration of PD-1^+^CD3^+^ T-cell frequency with these scores improved risk prediction, suggesting that immune phenotyping can complement organ dysfunction-based assessment. This is clinically meaningful because two patients with similar SOFA scores may have different immune states and therefore require different therapeutic strategies. However, the interpretation of PD-1^+^CD3^+^ T cells requires caution. PD-1 is not exclusively a marker of irreversible exhaustion; it can also be transiently expressed on recently activated T cells. Therefore, a single measurement may not fully distinguish physiological activation from pathological exhaustion. Dynamic monitoring may be more informative than one-time testing. For example, persistently elevated PD-1^+^CD3^+^ T cells during the later phase of sepsis may better indicate immune paralysis than early transient PD-1 upregulation during acute inflammation. Future studies should define optimal sampling time points, clinically relevant cut-off values, and whether changes in PD-1^+^CD3^+^ T-cell frequency can predict response to immune checkpoint blockade or other immunostimulatory therapies.

### 4.2 Soluble PD-1/PD-L1, IL-7 and lymphocyte dynamics

In addition to cellular checkpoint markers, soluble forms of PD-1 and PD-L1 may provide complementary information about systemic immune regulation in sepsis. Soluble PD-1 and soluble PD-L1 can be detected in peripheral blood and may reflect broader immune checkpoint activity across immune, endothelial, and tissue compartments. Unlike flow cytometry-based cellular markers, soluble markers are easier to measure using serum- or plasma-based assays and may be more feasible for repeated monitoring in critically ill patients. However, their biological interpretation can be complex because soluble checkpoint molecules may arise from multiple cellular sources and may not directly indicate the functional status of a specific T-cell subset.

Coman et al. examined the relationship among PD-1/PD-L1, IL-7, lymphocyte subsets, and disease progression (64). This work is useful because it places checkpoint molecules within the broader context of lymphocyte homeostasis rather than evaluating them as isolated markers. In sepsis, lymphocyte dynamics are highly relevant because persistent lymphopenia is a hallmark of immune suppression and is associated with impaired host defense. The relationship between PD-1/PD-L1 and lymphocyte subsets suggests that checkpoint activation and lymphocyte loss may interact to shape the immunosuppressive phenotype. IL-7 is particularly important in this context. As a key cytokine for T-cell survival, homeostatic proliferation, and functional maintenance, IL-7 is closely linked to adaptive immune recovery. Reduced or insufficient IL-7 signaling may contribute to defective T-cell reconstitution after sepsis-induced lymphopenia. Conversely, altered IL-7 levels may reflect a compensatory response to T-cell depletion. Therefore, IL-7 has dual significance: it may serve as a biomarker of impaired lymphocyte homeostasis and as a potential therapeutic molecule for immune restoration. The association between IL-7, PD-1/PD-L1, and lymphocyte dynamics suggests that immune stratification in sepsis should not rely on one marker alone. A combined panel including lymphocyte counts, PD-1/PD-L1-related markers, and IL-7 may better identify patients with reversible T-cell dysfunction.

From a clinical perspective, soluble PD-1/PD-L1 and IL-7 may be especially useful for longitudinal immune monitoring. Serial measurements could help determine whether a patient is progressing from inflammatory activation toward immune exhaustion, whether lymphocyte recovery is occurring, and whether immune-reversal therapy may be appropriate. However, further validation is needed to clarify whether these markers can guide treatment decisions, predict secondary infection, or identify patients who would benefit from IL-7-based or checkpoint-targeted interventions.

### 4.3 Peripheral blood metabolic biomarkers: GLS and dysfunctional CD4^+^ T-cell metabolism

Checkpoint-based biomarkers provide important information, but they do not fully capture the metabolic dimension of T-cell dysfunction. T-cell activation, proliferation, cytokine production, and survival require coordinated metabolic programs involving mitochondrial respiration, glycolysis, amino acid metabolism, and redox balance. In sepsis, these metabolic pathways are often disrupted by systemic inflammation, hypoxia, oxidative stress, nutrient fluctuation, and organ dysfunction (65–68). Therefore, metabolic biomarkers may help identify exhausted or dysfunctional T cells that cannot recover effective immune activity even when inhibitory receptor pathways are blocked.

From a biomarker perspective, GLS is important not because it introduces a separate mechanism, but because reduced GLS expression in CD4^+^ T cells may provide a measurable readout of impaired glutamine-dependent metabolic fitness. Yang et al. reported that diminished GLS expression in CD4^+^ T cells was associated with prognosis in septic patients (59). Thus, GLS may help identify patients whose T cells are metabolically compromised and may have limited capacity to recover proliferation and cytokine production even after checkpoint inhibition. At present, GLS should be regarded as a candidate metabolic biomarker rather than a validated therapeutic target. Larger cohorts, standardized detection platforms, and longitudinal sampling are required before GLS can be incorporated into clinical immune-monitoring panels. Metabolic biomarkers also provide a bridge between prognosis and therapy. More broadly, GLS and related metabolic indicators may complement checkpoint-based and lymphocyte-based markers in multi-parameter immune profiling. Such panels could integrate cellular exhaustion markers, soluble checkpoint molecules, lymphocyte counts, cytokine profiles, and metabolic readouts to define distinct immune states in septic patients, rather than relying only on inflammatory severity or organ dysfunction.

Overall, biomarkers of sepsis-induced T-cell exhaustion should move from single-marker assessment toward integrated immune profiling. PD-1^+^CD3^+^ T cells provide a cellular measure of checkpoint-associated T-cell dysfunction. Soluble PD-1/PD-L1 and IL-7 reflect systemic checkpoint activity and lymphocyte homeostasis. GLS adds a metabolic dimension by indicating impaired CD4^+^ T-cell bioenergetic fitness. Together, these markers may support more precise patient stratification, dynamic immune monitoring, and rational selection of immune-reversal strategies in sepsis.

## 5 Immune-reversal strategies: from checkpoint blockade to metabolic restoration

The recognition that sepsis is frequently followed by adaptive immune suppression has stimulated interest in immune-reversal strategies aimed at restoring protective host immunity. Unlike conventional anti-inflammatory therapies, immune-reversal approaches seek to rescue lymphocyte survival, improve T-cell effector function, enhance pathogen clearance, and reduce susceptibility to secondary infections. However, sepsis is highly dynamic. The same patient may experience hyperinflammation, immune paralysis, or mixed immune states at different disease stages. Therefore, immune-reversal therapy should not be viewed as indiscriminate immune stimulation. Instead, it requires careful consideration of timing, immune phenotype, infection control, and organ injury. Recent studies suggest that checkpoint blockade, cytokine-based immune restoration, and metabolic reprogramming may represent complementary strategies for reversing sepsis-induced T-cell exhaustion (69, 70).

### 5.1 PD-1/PD-L1 blockade: promise, timing and risk

PD-1/PD-L1 blockade is the most established immune-reversal strategy for exhausted T cells, largely because this pathway has been extensively studied in cancer immunotherapy and chronic infection (71, 72). In sepsis, PD-1 is commonly upregulated on dysfunctional T cells, while PD-L1 may be expressed by monocytes, macrophages, dendritic cells, neutrophils, endothelial cells, and tissue compartments exposed to inflammatory or microbial signals (15, 22, 33). The interaction between PD-1 and PD-L1 suppresses T-cell receptor signaling, limits proliferation, reduces cytokine production, and weakens antibacterial immunity (34). Therefore, blocking this axis may theoretically restore T-cell function and improve pathogen clearance in immunosuppressed septic patients. However, PD-1/PD-L1 blockade in sepsis cannot be directly modeled on cancer immunotherapy. In cancer, immune checkpoint blockade is usually intended to overcome tumor-induced immune tolerance in a relatively chronic disease context. In sepsis, the immune environment is acute, unstable, and often accompanied by tissue injury, organ dysfunction, and fluctuating microbial burden (33, 40). Therefore, three questions are central: when should PD-1/PD-L1 be blocked, which patients should receive blockade, and how can excessive inflammation or organ damage be avoided? In this regard, Xiang et al. provided direct evidence that the timing of PD-1/PD-L1 blockade influences organ protection during sepsis, supporting the concept that checkpoint inhibition should be considered within a defined therapeutic window rather than applied indiscriminately (40).

As discussed in Section 3.1, PD-1/PD-L1 signaling has stage-dependent functions during sepsis. From a therapeutic perspective, the key issue is not whether this pathway is immunosuppressive, but when and in whom it should be blocked. Consistent with this evidence, current data support a timing- and phenotype-guided approach, in which PD-1/PD-L1 blockade should be considered only when adaptive immune suppression predominates, while patients with uncontrolled hyperinflammation or ongoing organ injury should be treated with particular caution (40). Patient selection is equally important. Not all septic patients exhibit the same degree of T-cell exhaustion. Those with persistent lymphopenia, elevated PD-1^+^ T cells, high PD-L1 expression, reduced T-cell cytokine production, or recurrent secondary infections may be more suitable candidates for checkpoint-targeted therapy. In contrast, patients dominated by uncontrolled hyperinflammation, severe cytokine storm, or ongoing tissue injury may not benefit and could potentially be harmed. This supports a biomarker-guided model in which PD-1/PD-L1 blockade is reserved for patients with demonstrable adaptive immune suppression. EV-associated PD-L1, discussed mechanistically in Section 3.1, may also have therapeutic relevance as a candidate biomarker or target. However, EV-PD-L1-directed intervention remains an early concept and should be presented as a future direction rather than a validated immune-reversal strategy.

### 5.2 TIGIT blockade as an emerging immune-restorative strategy

TIGIT blockade represents an emerging immune-reversal strategy in sepsis (16, 44). TIGIT is an inhibitory receptor expressed on activated T cells, regulatory T cells, natural killer cells, and other lymphocyte subsets. By interacting with ligands such as CD155 and CD112, TIGIT can suppress T-cell activation, reduce cytokine production, and promote immune tolerance (73, 74). Although TIGIT has been more extensively studied in cancer, recent evidence suggests that it may also contribute to adaptive immune dysfunction during sepsis (44). As discussed in Section 3.2, TIGIT functions as an inhibitory regulator of CD4^+^ T-cell antibacterial immunity during polymicrobial sepsis. Therefore, TIGIT blockade may represent a potential immune-restorative strategy, particularly for patients with TIGIT-dominant checkpoint activation. However, its therapeutic use remains experimental and will require careful validation of timing, dosing, and safety (21, 22). Therapeutically, TIGIT blockade may be especially relevant for restoring CD4^+^ T-cell-mediated antibacterial immunity. CD4^+^ T cells are essential for coordinating macrophage activation, supporting B-cell responses, regulating cytokine networks, and shaping innate–adaptive crosstalk. If TIGIT suppresses these functions during sepsis, its blockade may help restore immune coordination rather than simply increasing T-cell activation. TIGIT may also interact with PD-1/PD-L1 signaling, forming a broader inhibitory checkpoint network. Therefore, future studies should determine whether TIGIT blockade is most effective alone, in combination with PD-1/PD-L1 blockade, or in selected patients with TIGIT-dominant immune suppression. As with PD-1/PD-L1 inhibition, timing and patient selection will be critical to avoid excessive immune activation.

### 5.3 IL-7-based immune restoration

Cytokine-based immune restoration provides another strategy for reversing sepsis-induced T-cell dysfunction (21, 75). Interleukin-7 (IL-7) is a key homeostatic cytokine required for T-cell survival, proliferation, and functional maintenance (76). It supports naïve and memory T-cell homeostasis, promotes anti-apoptotic signaling, and may improve lymphocyte recovery after sepsis-induced depletion (76, 77). Because persistent lymphopenia is a major feature of sepsis-associated immune paralysis, IL-7 has attracted attention as a potential immune-restorative therapy. As discussed in Section 4.2, IL-7 is closely linked to lymphocyte homeostasis and adaptive immune recovery during sepsis (64). Thus, IL-7 has dual significance as both a marker of lymphocyte homeostasis and a candidate molecule for immune restoration. IL-7-based therapy may be particularly suitable for patients with persistent lymphopenia, reduced T-cell proliferation, and evidence of adaptive immune suppression (75, 78). Unlike checkpoint blockade, which primarily releases inhibitory signaling, IL-7 may rebuild the T-cell compartment by enhancing survival and homeostatic expansion. This distinction is important because some septic patients may have too few functional T cells for checkpoint blockade alone to be effective. In such cases, IL-7 could potentially complement checkpoint-targeted approaches. However, IL-7 therapy also requires careful immune monitoring, because restoring lymphocyte activity in an uncontrolled inflammatory environment could theoretically amplify harmful immune responses (21, 22). Despite these encouraging immune-restorative effects, IL-7 should not be presented as a risk-free intervention. Because IL-7 promotes T-cell survival, proliferation, and functional recovery, its long-term consequences in sepsis remain incompletely defined. Potential concerns include excessive or prolonged T-cell expansion, persistence of inflammatory activation, exacerbation of subclinical autoimmunity, altered immune homeostasis after recovery, and uncertain effects on susceptibility to recurrent or secondary infections. In addition, the route of administration may influence safety. Although intramuscular CYT107 was well tolerated in patients with septic shock and severe lymphopenia, intravenous CYT107 was associated with transient fever and respiratory distress in some patients, supporting the need for careful dose, route, timing, and patient-selection strategies (75, 78). Therefore, future IL-7 trials should include extended follow-up, longitudinal immune monitoring, and systematic assessment of delayed inflammatory, autoimmune, infectious, and organ-related adverse events.

### 5.4 Metabolic reprogramming as an immune-reversal strategy

T-cell exhaustion in sepsis is not only a checkpoint problem; it is also a metabolic problem (79, 80). T-cell activation and effector function require mitochondrial fitness, glycolytic flexibility, amino acid metabolism, redox control, and adequate biosynthetic capacity (56, 81, 82). Sepsis disrupts these programs through hypoxia, oxidative stress, nutrient fluctuation, inflammatory cytokines, organ dysfunction, and systemic catabolism. Therefore, even if inhibitory receptors are blocked, metabolically exhausted T cells may remain unable to proliferate or produce effective cytokine responses. This provides the rationale for metabolic restoration as an immune-reversal strategy.

As discussed in Section 3.4, post-septic CD4^+^ T cells may exhibit maladaptive metabolic remodeling rather than productive immune activation. Therefore, metabolic restoration should aim to improve coordinated bioenergetic fitness rather than broadly stimulate T-cell activity. Potential metabolic interventions may include improving mitochondrial function, correcting the balance between glycolysis and oxidative phosphorylation, supporting redox homeostasis, and restoring amino acid metabolism (56, 57, 81). Importantly, metabolic restoration should not be limited to glutamine metabolism alone. Future interventions may also need to consider arginine and tryptophan metabolism, lactate accumulation, tricarboxylic acid cycle remodeling, lipid metabolic flexibility, and the interaction between nutrient availability and checkpoint signaling. However, these approaches remain exploratory and should be guided by validated immune-metabolic biomarkers rather than by broad or non-specific metabolic stimulation. GLS-related metabolic insufficiency is discussed as a biomarker in Section 4.3. From a therapeutic perspective, this finding suggests that future metabolic interventions should be guided by validated immune-metabolic profiles rather than by broad nutritional or metabolic stimulation.

Targeting dysregulated IL-17 responses may also be relevant. Post-septic CD4^+^ T cells may produce abnormal IL-17 responses that fail to protect against secondary infection and may contribute to inflammatory imbalance (56). Rather than broadly suppressing or enhancing IL-17, future studies should clarify whether IL-17 dysregulation reflects pathogenic inflammation, failed antimicrobial defense, or maladaptive differentiation of CD4^+^ T cells. This distinction will be important before IL-17-related pathways can be therapeutically targeted. Overall, immune-reversal strategies in sepsis should move toward integrated and personalized approaches. PD-1/PD-L1 blockade may release inhibitory checkpoint signaling, TIGIT blockade may restore CD4^+^ T-cell antibacterial immunity, IL-7 may promote lymphocyte survival and expansion, and metabolic reprogramming may improve the bioenergetic fitness required for sustained immune recovery (16, 22). The next challenge is to match each strategy to the appropriate patient, immune phenotype, and disease stage. A rational model would combine cellular markers, soluble checkpoint molecules, lymphocyte counts, cytokine profiles, and metabolic biomarkers to guide when and how to reverse T-cell exhaustion without aggravating inflammatory organ injury.

## 6 Challenges and future perspectives

Despite growing evidence that T-cell exhaustion contributes to sepsis-induced immune paralysis, several conceptual and translational challenges remain (21, 22). First, sepsis-induced T-cell exhaustion should not be considered identical to the exhaustion programs described in cancer or chronic viral infection. In tumors and chronic viral infection, T-cell exhaustion usually develops under persistent antigen stimulation and is accompanied by relatively stable transcriptional and epigenetic remodeling (18, 83). In contrast, sepsis is an acute and highly dynamic syndrome characterized by rapidly changing pathogen burden, systemic inflammation, tissue injury, lymphocyte apoptosis, organ dysfunction, metabolic stress, and exposure to antimicrobial or supportive therapies (21, 84). These factors may generate an exhaustion-like T-cell state that overlaps phenotypically with classical exhaustion but differs mechanistically. The recent study showing that TOX does not drive sepsis-induced T-cell exhaustion is especially important in this context (47). Although septic T cells may upregulate PD-1, LAG-3, TIM-3, and other inhibitory receptors, their dysfunctional state may not depend on the canonical TOX-centered exhaustion program (47). Therefore, future research should define sepsis-specific transcriptional and epigenetic circuits rather than simply applying models derived from oncology or chronic infection.

Second, dynamic immune monitoring is likely more informative than single-time-point biomarker assessment (22, 64). Many proposed markers of T-cell dysfunction, including PD-1, PD-L1, TIGIT, IL-7, GLS, and lymphocyte counts, may change substantially across the disease course (16, 58, 64). A biomarker measured at ICU admission may reflect acute immune activation, whereas the same marker measured several days later may indicate persistent immune suppression. For example, PD-1 expression may represent transient activation in early infection but pathological exhaustion if it remains elevated during the immunosuppressive phase (15, 22). Similarly, IL-7 may reflect compensatory lymphocyte homeostasis, while reduced GLS may indicate impaired metabolic recovery of CD4^+^ T cells (58, 64). The studies evaluating PD-1/PD-L1 with IL-7 dynamics and PD-1^+^CD3^+^ T cells as mortality predictors support the value of immune phenotyping but also highlight the need for longitudinal sampling (63, 64). Future studies should develop immune trajectories rather than static cut-off values, integrating early, intermediate, and late time points to identify patients entering a reversible immunosuppressive state.

Third, patient stratification is essential for immune-reversal therapy (21, 22). Sepsis is biologically heterogeneous, and not all patients will benefit from the same immunomodulatory strategy. Immune checkpoint blockade may be beneficial in patients with persistent lymphopenia, high PD-1^+^ or TIGIT^+^ T-cell frequencies, reduced cytokine production, recurrent secondary infection, or evidence of impaired bacterial clearance (15, 16, 27). However, the same intervention may be ineffective or harmful in patients dominated by uncontrolled hyperinflammation, severe cytokine storm, or ongoing tissue injury (21, 22). The study on the optimal timing of PD-1/PD-L1 blockade illustrates this point clearly: therapeutic benefit depends not only on the pathway being targeted but also on when it is targeted (40). Therefore, future clinical translation should prioritize biomarker-guided intervention windows. Instead of treating all septic patients with broad immune stimulation, trials should stratify patients according to T-cell phenotype, checkpoint expression, cytokine profile, metabolic state, microbial burden, and organ dysfunction.

Fourth, integrated single-cell, spatial, and functional immunology will be needed to move the field beyond descriptive markers (11, 21). Peripheral blood analysis is clinically accessible, but it may not fully reflect immune events within infected tissues, lymphoid organs, or sites of organ injury. Single-cell RNA sequencing can define heterogeneous T-cell states, while T-cell receptor repertoire analysis can reveal clonal expansion, antigen experience, and repertoire collapse (11, 21). Mass cytometry or high-dimensional flow cytometry can quantify co-expression patterns of PD-1, TIGIT, TIM-3, LAG-3, 2B4, activation markers, cytokines, and metabolic proteins at the single-cell level. Spatial transcriptomics and multiplex imaging may help determine whether exhausted or dysfunctional T cells localize near infected tissues, endothelial barriers, myeloid-rich niches, or damaged organs. In parallel, metabolic flux analysis and ex vivo T-cell stimulation assays are needed to distinguish phenotypic exhaustion from true functional impairment (55). Combining longitudinal immune monitoring with functional assays may allow researchers to determine whether a patient’s T cells are merely checkpoint-positive, metabolically impaired, apoptotic, anergic, or genuinely reversible.

Another limitation is that the metabolic discussion in this review mainly focuses on CD4^+^ T cells. This choice reflects the availability of direct evidence linking CD4^+^ T-cell metabolic remodeling to post-septic immune dysfunction, but it does not fully capture the metabolic heterogeneity of other T-cell subsets or immune populations. CD8^+^ T cells, regulatory T cells, γδ T cells, natural killer cells, monocytes, macrophages, and dendritic cells may undergo distinct metabolic adaptations during sepsis. These changes may influence cytotoxic activity, immune regulation, antigen presentation, cytokine production, and susceptibility to secondary infection. Future studies should therefore compare metabolic programs across immune-cell subsets and determine how subset-specific metabolic failure contributes to sepsis-induced immune paralysis.

Looking forward, the most important challenge is to build an integrated model of sepsis-induced T-cell exhaustion that links phenotype, mechanism, timing, and therapeutic response (21, 22). Such a model should recognize that T-cell exhaustion in sepsis is not a fixed endpoint but a dynamic immune state shaped by infection, inflammation, tissue injury, and metabolic stress. Future studies should identify which exhausted or dysfunctional T-cell states are reversible, which biomarkers best predict recovery, and which immune-reversal strategies can restore host defense without worsening inflammatory organ damage.

## 7 Conclusion

T-cell exhaustion is an important mechanism underlying sepsis-induced immune paralysis. Although sepsis has traditionally been viewed as a hyperinflammatory syndrome, many patients develop a later or concurrent immunosuppressive state characterized by lymphopenia, impaired T-cell proliferation, reduced effector cytokine production, increased susceptibility to secondary infection, and poor immune recovery. Within this process, exhausted or dysfunctional T cells show increased expression of inhibitory receptors, altered cytokine profiles, metabolic reprogramming, and weakened antibacterial activity. Recent studies have refined this concept by identifying several emerging mechanisms, as discussed above. TIGIT has been shown to suppress CD4^+^ T-cell antibacterial immunity in polymicrobial sepsis, suggesting that the checkpoint landscape extends beyond PD-1/PD-L1. Extracellular vesicle-associated PD-L1 provides a mechanism by which systemic checkpoint-mediated immunosuppression may spread beyond direct cell–cell contact. Qin et al. further indicates that septic T-cell dysfunction is not simply a copy of classical exhaustion in cancer or chronic viral infection (47). In addition, metabolic abnormalities, including mitochondrial dysfunction, dysregulated IL-17 production, enhanced glycolytic remodeling, and reduced GLS expression in CD4^+^ T cells, suggest that bioenergetic failure is a key component of impaired T-cell recovery. These insights also have translational implications. PD-1^+^CD3^+^ T cells, PD-1/PD-L1-related markers, IL-7, TIGIT, EV-PD-L1, and GLS may contribute to immune stratification and prognostic assessment. However, no single marker is likely to fully capture the complexity of sepsis-induced T-cell exhaustion. Future approaches should combine cellular phenotyping, soluble biomarkers, metabolic indicators, functional assays, and longitudinal monitoring.

Immune-reversal strategies should therefore be precise rather than broadly stimulatory. PD-1/PD-L1 blockade, TIGIT inhibition, IL-7-based immune restoration, and metabolic reprogramming may all have therapeutic potential, but their success will depend on patient selection, timing, infection control, and organ injury status. A personalized and dynamically monitored approach may ultimately allow restoration of protective immunity while minimizing the risk of exacerbating harmful inflammation.
